# Supplementary material for: Glucose Starvation or Pyruvate Dehydrogenase Activation Induce a Broad, ERK5-Mediated, Metabolic Remodeling Leading to Fatty Acid Oxidation
Source: Cells. 2022 Apr 20;11(9):1392. doi: 10.3390/cells11091392 (PMC9104157; doi:10.3390/cells11091392)
Supplement: Supplementary file 1 [file cells-11-01392-s001.zip › cells-1663494-supplementary.pdf]

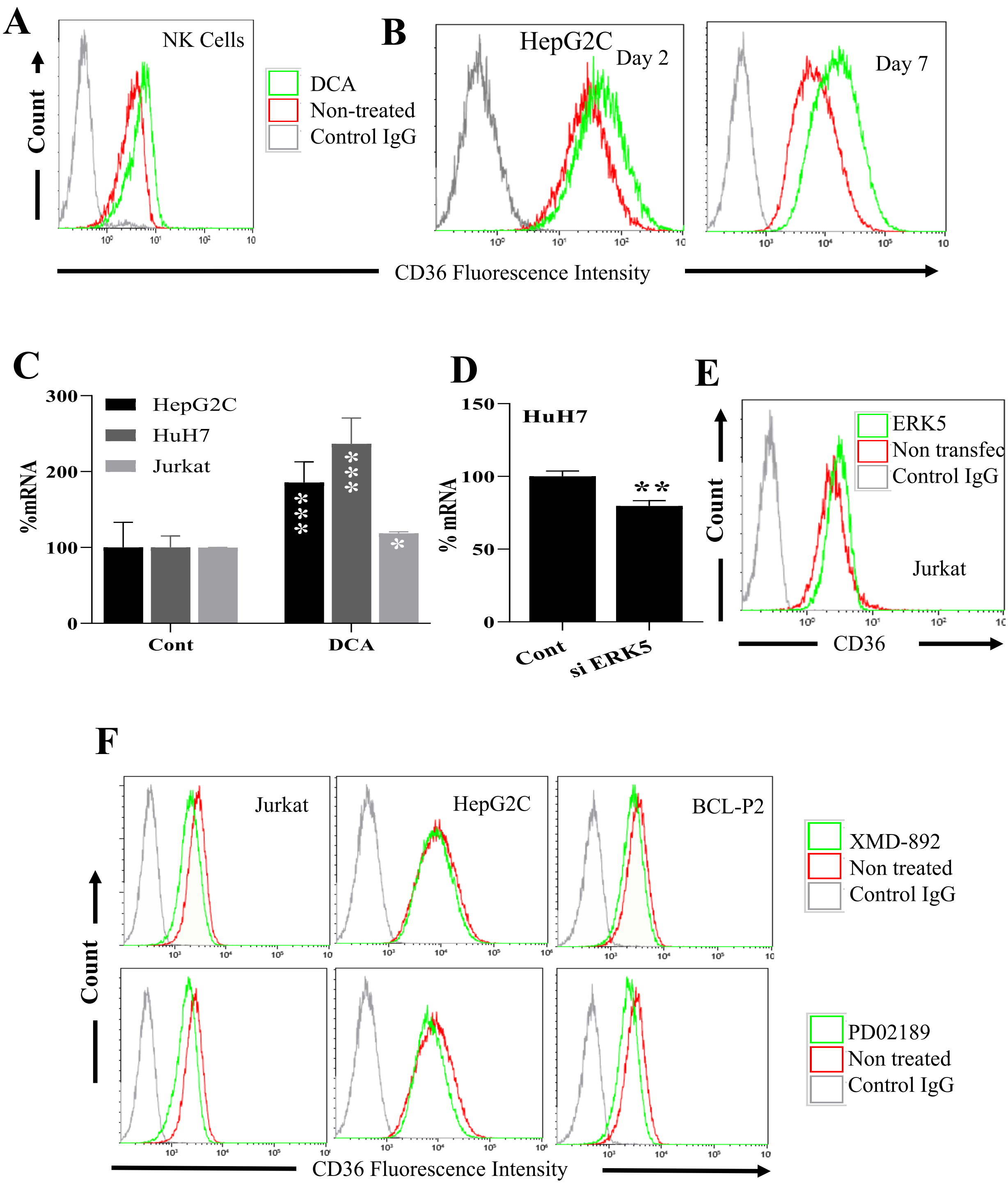

Figure S1. Metabolic changes regulate CD36 expression through MEK5/ERK5 pathway. A-C) *CD36* mRNA or CD36 membrane protein were analyzed in different cell types treated with 5 mM DCA for 3 days or as indicated in (B). **D-E)** Different cell lines were treated with siRNA for ERK5 or with the ERK5 inhibitor XMD-892 (5 µM) or the MEK5 inhibitor PD02189 (5 µM) for 24 hours and *CD36* mRNA (D) or protein (E) were analyzed. F) Jurkat cells were transfected with an ERK5 expression plasmid and 3 days later CD36 expression was analyzed. Bar graphs represent means ± SD of at least 3 independent experiments performed in triplicate. * p<0.05, ** p<0.01, *** p<0.005 compare to control cells.

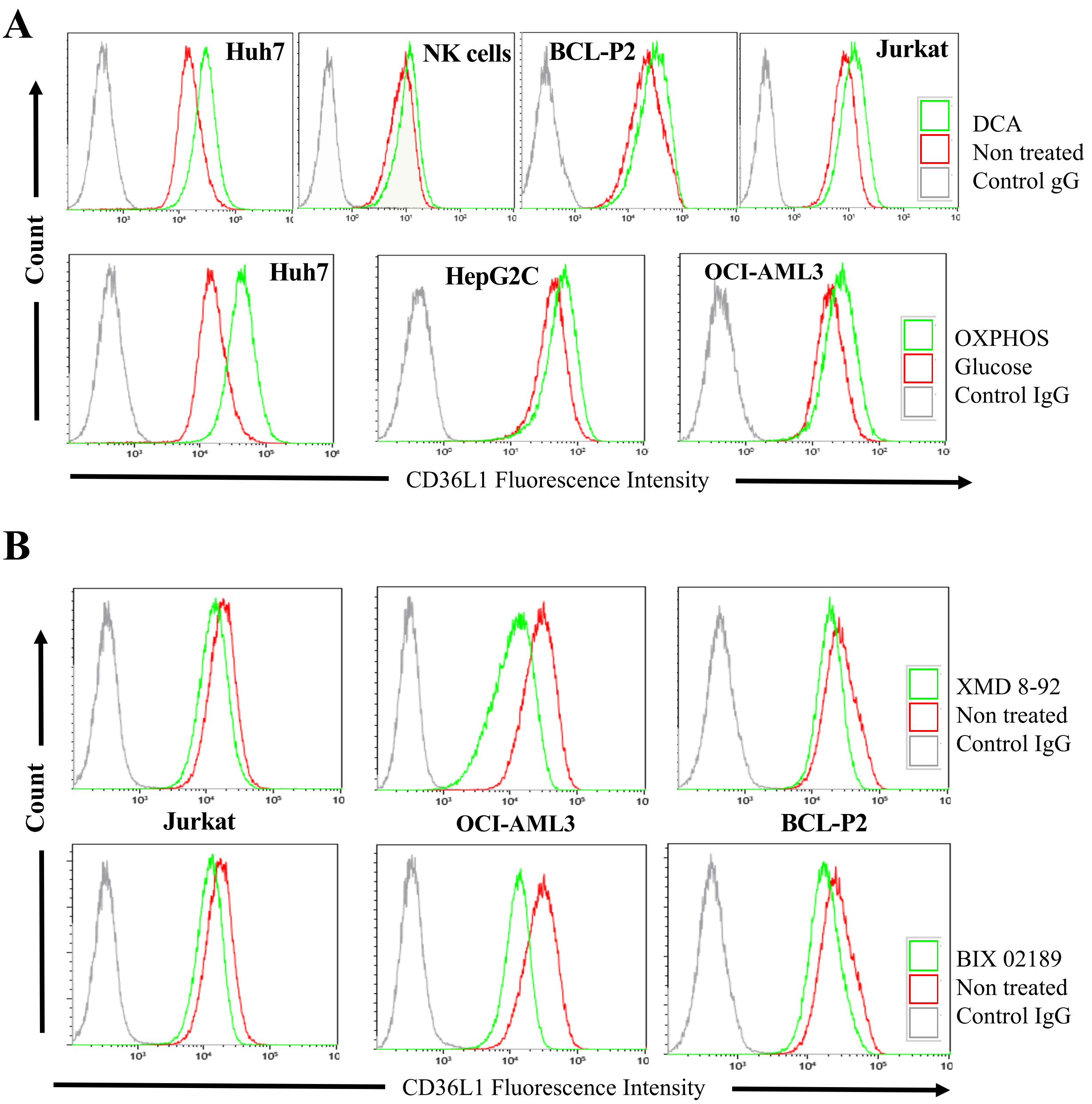

Figure S2. Changes in cell metabolism regulate SCARB1/CD36L1 expression. CD36L membrane protein was analyzed in different cell types treated for 3 days with DCA (5 mM) or growing in a free-glucose medium for 5-7 days (A) or treated with the ERK5 (XMD 892 10 µM) or with the MEK5 (BIX 02189 5 µM) inhibitors for 24 h (B).

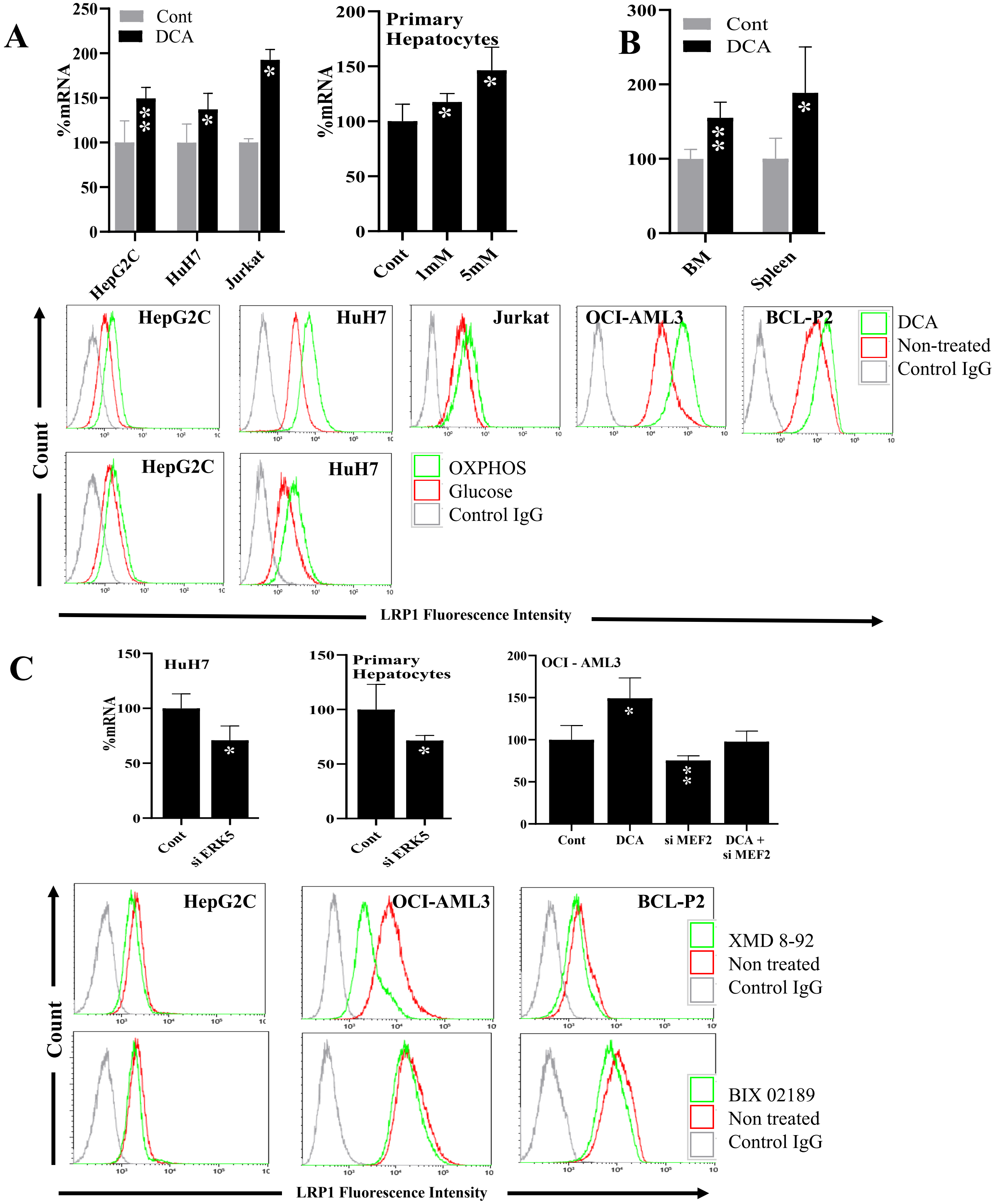

Figure S3. Metabolic changes regulate LRP1 expression through the MEK5/ERK5 pathway. A) *LRP1* mRNA (up) and LRP1 membrane protein (low) were analyzed in different cell types treated with DCA (5 mM) or growing in a free-glucose medium for 5-7 days. B) NSG mice were engrafted with primary human AML cells. At day 80 post-graft, they were treated with DCA (n = 4) or leave untreated (n = 4). At day 140, mRNA from bone marrow or spleen was isolated and human *LRP1* mRNA expression was quantified by qPCR. C) *LRP1* mRNA (up) and LRP1 membrane protein (low) were analyzed in different cell types treated with siRNA for ERK5 or for MEF2 or treated with the ERK5 (XMD 892 (10 µM) or with the MEK5 BIX 02189 (5 µM) inhibitors for 24 h. Bar graphs represent means ± SD of at least 3 independent experiments performed in triplicate. * $p<0.05$, ** $p<0.01$, compare to control cells.

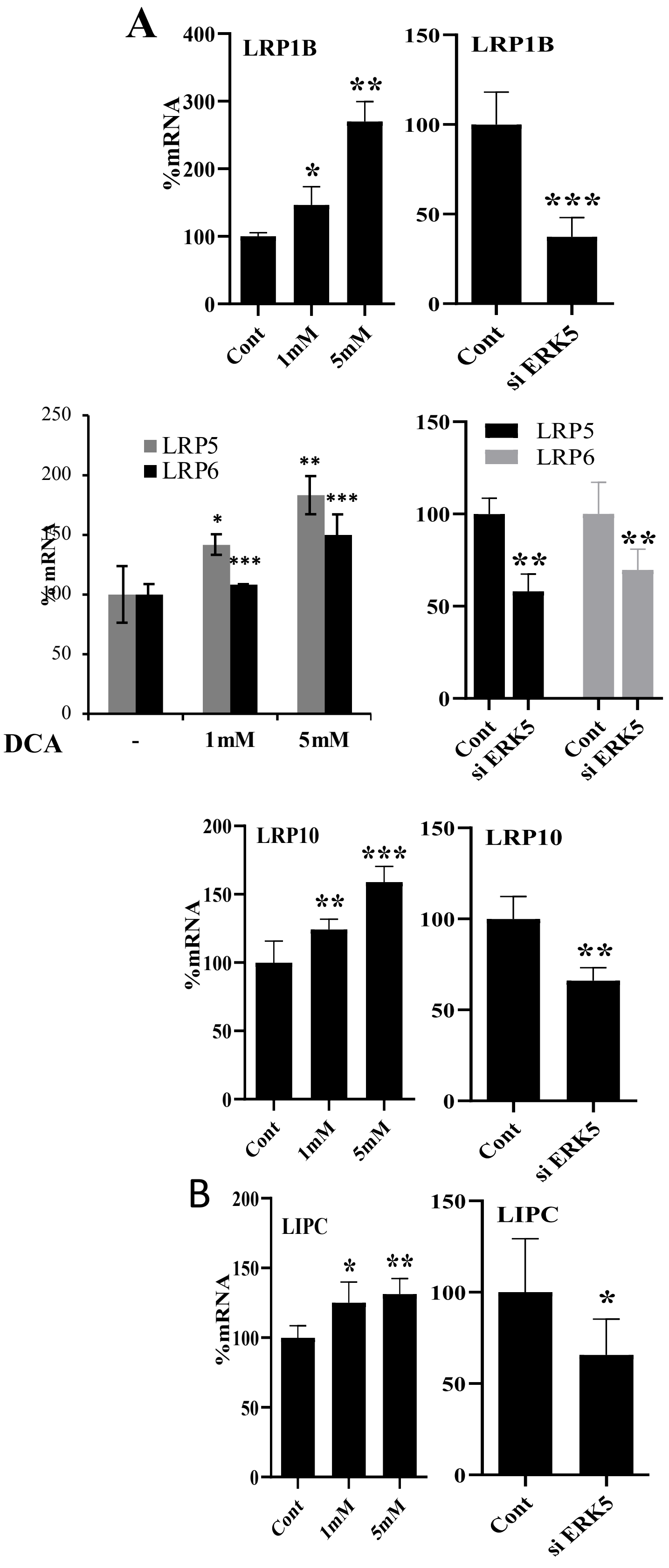

Figure S4. **Metabolic changes regulate LRP1B, LRP5/6, LIRP10, and LIPC expression through the MEK5/ERK5 pathway.** The *LRP1B*, *LIRP10, LRP5/6 (A)* and *LIPC(B)* mRNA was analyzed in primary hepatocytes treated with the indicated doses of DCA for 48h or transfected with siRNA of ERK5. Bar graphs represent means ± SD of at least 3 independent experiments performed in triplicate. * $p<0.05$, ** $p<0.01$, *** $p<0.005$ compare to control cells.

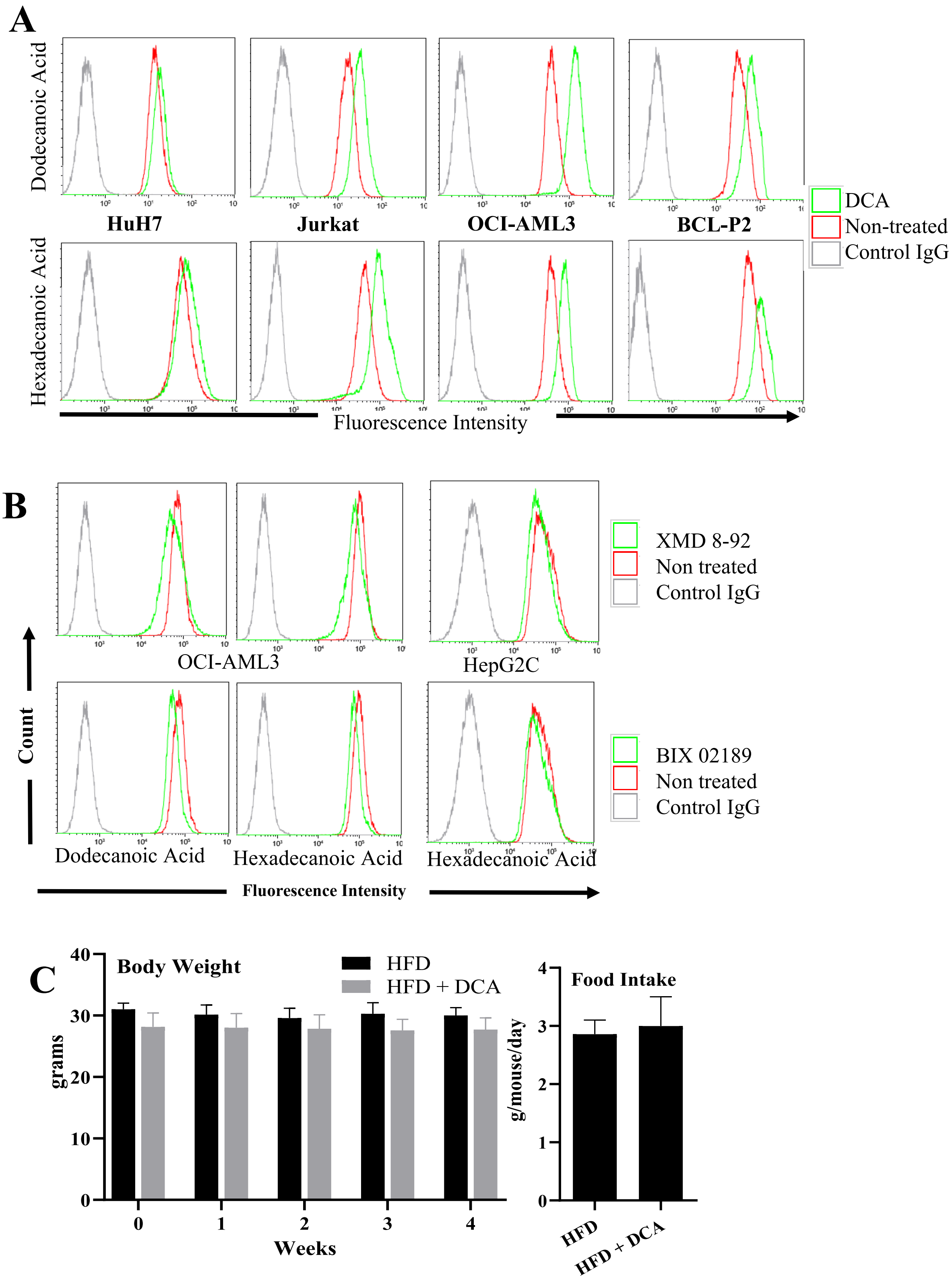

Figure S5. Metabolic changes regulate FA transport through the MEK5/ERK5 pathway. A) Fluorescent hexadecanoic and dodecanoic acid transport was analyzed in different cell types treated with DCA (5 mM for 5 days). B) Cells were treated with ERK5 (10 µM) and MEK5 (5 µM) inhibitors for 24 h before analyzing FA transport. **C)** DCA treatment does not affect body weight or food intake in mice. Mice were treated with DCA (50 mg/kg per day) for four weeks. Bar graphs represent means ± SD of at least 3 independent experiments performed in triplicate.

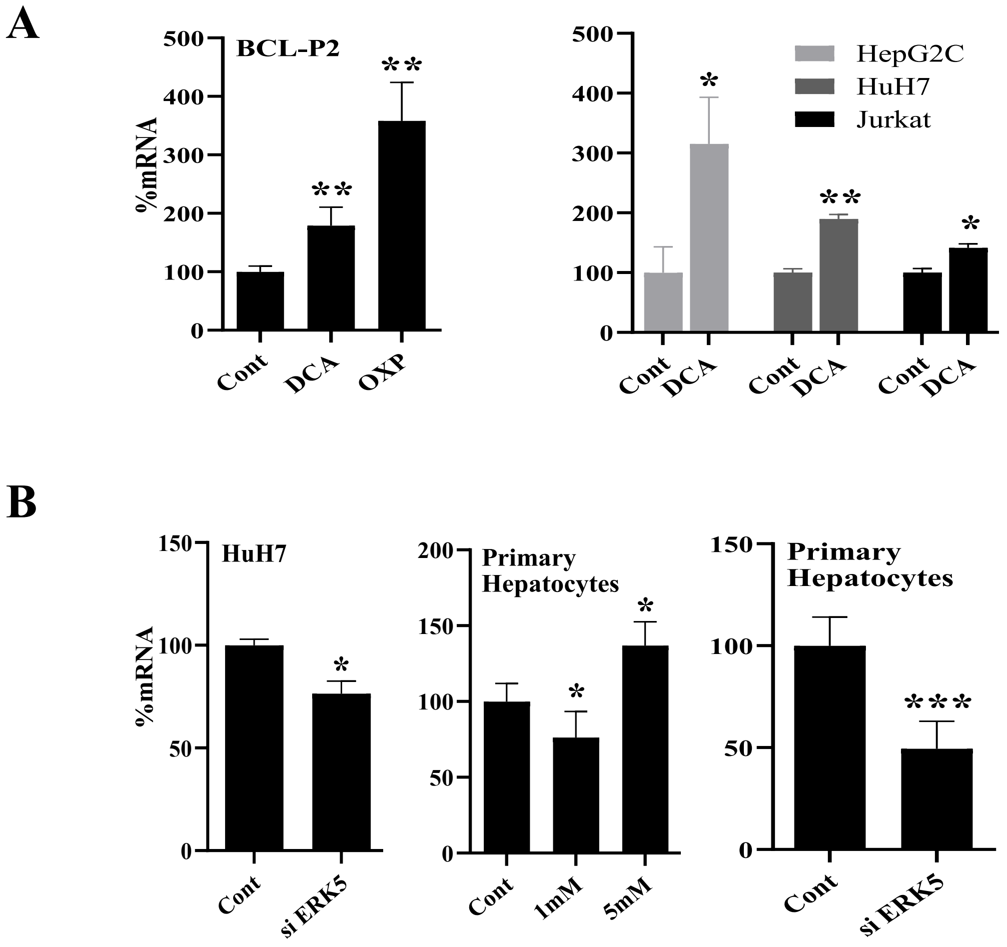

Figure S6. Metabolic changes regulate the expression of the ACSL family of ligases through the MEK5/ERK5 pathway. **A)** The *ACSL1* and **B)** *ACSL6* mRNA was analyzed in different cell types treated with 5 mM DCA for 3 days or growing in a free-glucose medium that induced OXPHOS for 5-7 days or in cells treated for 72 h with siRNA for ERK5 or MEF2. Bar graphs represent means ± SD of at least 3 independent experiments performed in triplicate. * $p<0.05$, ** $p<0.01$, *** $p<0.005$ compare to control cells.

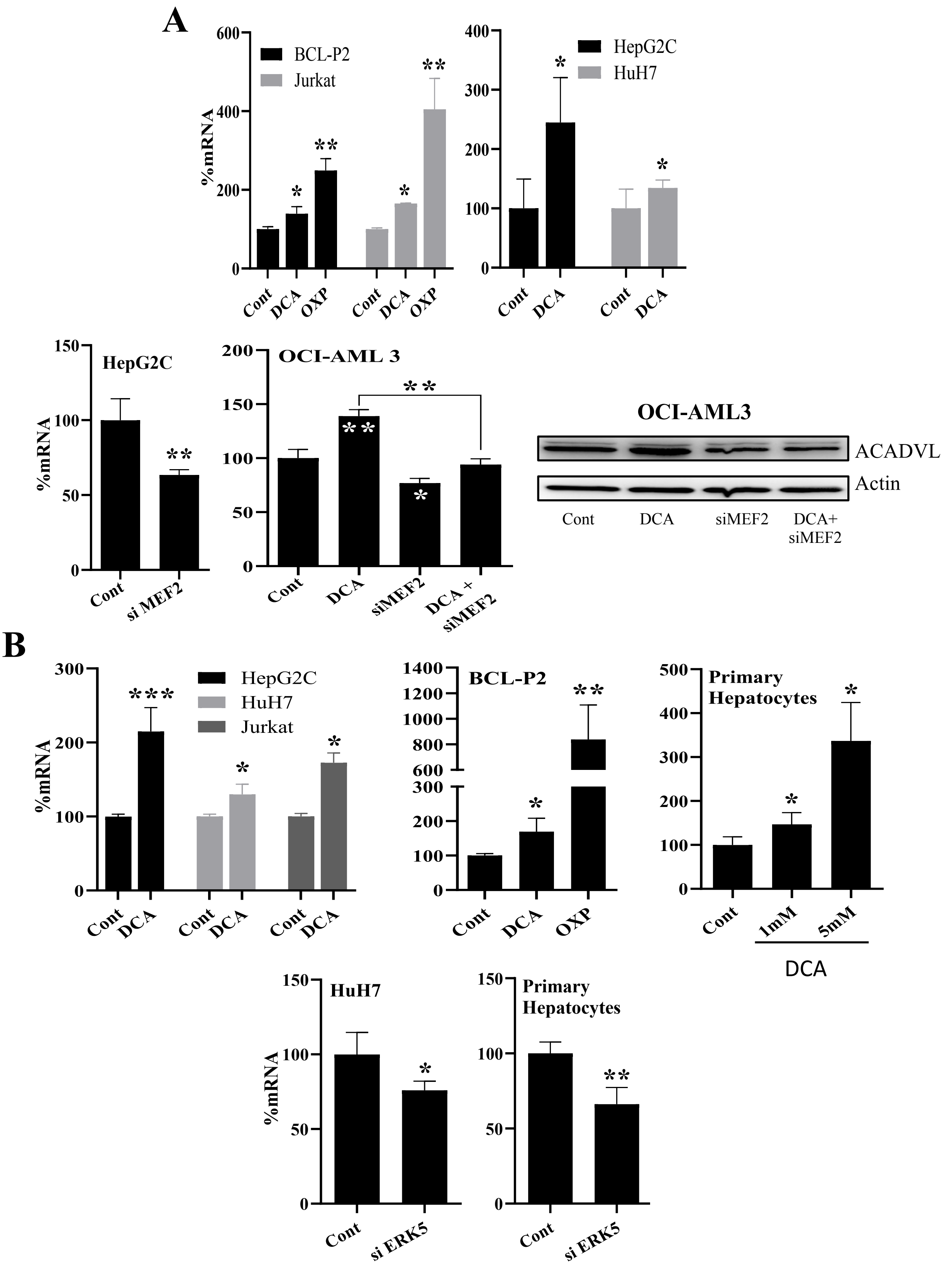

Figure S7. **Metabolic changes regulate the expression of the acyl-CoA dehydrogenases through the MEK5/ERK5 pathway. A)** The *ACADVL1* and **B)** *ACADM* mRNA or protein were analyzed in different cell types treated with DCA or growing in a free-glucose medium for 5-7 days or in cells treated with siRNA for ERK5 or MEF2 for 72 hours. Bar graphs represent means ± SD of at least 3 independent experiments performed in triplicate. * p<0.05, ** p<0.01, *** p<0.005 compare to control cells or as depicted.
